# Implementation of an Organization-Based Couples Health Promotion Program to Improve Physician Well-Being

**DOI:** 10.1001/jamanetworkopen.2025.3218

**Published:** 2025-04-04

**Authors:** Jessica M. Gold, Tait D. Shanafelt, Hanhan Wang, Jo Townson, Sherilyn Stolz, Nikitha Menon, Mickey Trockel

**Affiliations:** 1 Department of Pediatrics, Stanford University School of Medicine, Stanford, California; 2 Department of Internal Medicine, Stanford University School of Medicine, Stanford, California; 3 Stanford University School of Medicine, Stanford, California; 4 Department of Psychiatry and Behavioral Sciences, Stanford University School of Medicine, Stanford, California

## Abstract

**Importance:**

Physicians work long, often unpredictable hours and experience multiple work-related stressors, which may adversely affect their personal relationships.

**Objective:**

To assess the associations of a couples’ workshop for physicians and their partners with burnout, self-valuation, and impact of work on personal relationships (IWPR).

**Design, Setting, and Participants:**

In this cohort study, participants were grouped into an immediate intervention group and a delayed intervention control group, which were later compared. The participants in the intervention group gathered at a resort near Stanford University in October 2022, whereas the participants in the control group were invited to participate in a May 2023 workshop. Invited participants included Stanford Medicine physicians and their partners. Eligible physicians worked in a department or division considered frontline during the COVID-19 pandemic or had unfavorable IWPR scores on an institution-wide survey. The data were analyzed from June 14, 2024, to October 1, 2024.

**Intervention:**

The intervention group participated in a 2-day workshop for couples and were offered 3 evening sessions. All sessions emphasized positive psychology principles and incorporated didactic, reflection, and unstructured content focused on strengthening relationships.

**Main Outcomes and Measures:**

The main outcome was IWPR score, measured at baseline (immediately preceding the workshop for the intervention group and at registration for the control group) and 6 months later. IWPR was measured with a survey asking 4 questions about how work had affected personal relationships during the past year, and the responses included not at all true, somewhat true, moderately true, very true, and completely true (with scores assigned as 0, 1, 2, 3, and 4, respectively). Other outcome measures included burnout and self-valuation (with a lower score being favorable for burnout and a higher score being favorable for self-valuation).

**Results:**

Of 47 intervention group participants and 69 control group participants, 22 (46.8%) and 40 (57.9%), respectively, were women (*P* = .32). Paired assessments (at baseline and 6 months) were completed by 38 of 47 (80.9%) physicians in the intervention group and 53 of 69 (76.8%) in the control group. Between baseline and 6-month follow-up, participants in the intervention group showed a mean (SD) improvement of 1.59 (2.66) points (Cohen *d* = 0.54 [95% CI, 0.23-0.85]; *P* < .001) in IWPR, 1.22 (1.47) points (Cohen *d* = 0.68 [95% CI, 0.39-0.98]; *P* < .001) in burnout, and –1.25 points (2.09) (Cohen *d* = 0.68 [95% CI, –0.97 to –0.25]; *P* < .001) in self-valuation. Statistically significant changes in these measures were not observed in the control participants. In mixed-effects modeling comparing the intervention and control groups, being in the intervention group was associated with a 1.25-point improvement in IWPR (Glass *d* = −0.45 [95% CI, −0.12 to −0.79]; *P* = .01), a 1.24-point improvement in burnout (Glass *d* = −0.70 [95% CI, −0.37 to −1.03]; *P* < .001), and a 0.97-point improvement in self-valuation (Glass *d* = 0.47 [95% CI, 0.09-0.86]; *P* = .02).

**Conclusions and Relevance:**

In this cohort study of an organizationally sponsored intervention consisting of a couples’ workshop designed to mitigate the adverse IWPR for physicians, participation was associated with statistically significant improvements in IWPR, burnout, and self-valuation. Further development of interventions to mitigate adverse IWPR among physicians is warranted.

## Key Points

**Question:**

Is a 2-day couples’ workshop for physicians and their partners associated with a reduction in the adverse impact of work on personal relationships (IWPR)?

**Findings:**

This cohort study of 47 intervention group participants and 69 control group participants found that, between baseline and 6-month follow-up, participants in the intervention group showed a mean improvement of 1.59 points in IWPR, 1.22 points in burnout, and 1.25 points in self-valuation and that significant changes in these measures were not observed in the control participants.

**Meaning:**

This study found that an organizationally sponsored intervention to mitigate adverse IWPR showed statistically significant improvements in IWPR and burnout.

## Teaser

This cohort study assesses the associations of a couples’ workshop for physicians and their partners with burnout, self-valuation, and impact of work on personal relationships.

## Introduction

In 2023, the US Surgeon General released a call to action entitled “Our Epidemic of Loneliness and Isolation.”^1^ The report details the myriad ways in which our social networks—which include our relationships with loved ones, families, friends, colleagues, and communities—provide support, structure, and numerous health benefits. The report also details the erosion of these networks over time and our increasing social isolation. Physicians may be at particular risk of having strained personal relationships, given long and often unpredictable work hours and a professional culture that values service and self-sacrifice.^2,3,4,5,6^ Extensive research indicates physicians experience greater struggles with work-life integration than US workers in other fields, a finding that persists after controlling for demographic factors, work hours, and level of education.^7,8,9^ Furthermore, work-life conflict is a well-established contributor to burnout and is cited as a frequent reason to scale back on work or leave medicine altogether.^10,11,12,13^

From 2022 to 2024, 3 studies specifically explored the impact of work on personal relationships (IWPR). Trockel et al^14^ found that physicians commonly experience an adverse IWPR and are more likely than workers in other fields to express that their jobs led to them feeling disconnected from their loved ones. Notably, Trockel et al,^15^ in a longitudinal study, found that physicians who reported that work adversely impacted their personal relationships were more likely to have unsolicited patient complaints over the ensuing 4 years. In that study, the adverse IWPR at baseline was more robustly associated with future patient complaints than other common well-being measures, including depression, anxiety, sleep-related impairment, burnout, and low professional fulfillment.^15^ Ligibel et al^16^ showed an adverse IWPR is also associated with the intent to leave clinical practice, even after adjusting for burnout and professional fulfillment.

Although these studies have provided insights into the association between IWPR and deleterious outcomes,^14,15^ there have been few published studies of interventions related to this domain. Employer-funded physician coaching programs have offered the opportunity for physicians to focus on work-life integration and personal relationships and have shown efficacy in improving burnout and professional fulfilment.^17,18^ One study showed modest improvement in relationship self-efficacy when health care professionals participated in a brief workshop related to work-home conflict.^19^ Although indirectly related to IWPR, these studies have focused on fostering work-life integration. To our knowledge, no published organizational interventions have involved the physician participating with their spouse or partner, nor specifically targeted the adverse IWPR, beyond work-home conflict. Here, we report the association of a novel couples’ workshop for physicians and their partners with physicians’ IWPR.

## Methods

In this cohort study, we conducted an organization-sponsored pilot intervention consisting of a weekend-long couples’ workshop for faculty physicians and their partners intended to mitigate the adverse IWPR. Use of program data for research purposes was reviewed by the Stanford University institutional review board and deemed exempt because it involved retrospective analysis of administratively collected data using a completely anonymized dataset. The Strengthening the Reporting of Observational Studies in Epidemiology (STROBE) reporting guideline for cohort studies was followed.^20^

### Setting

The Couples’ Health Promotion Program was created by the WellMD & WellPhD Center at Stanford University, an academic medical center. Eligible participants included Stanford Medicine physician faculty and their partners. The mainstay of the intervention included an organizationally sponsored 2-day workshop at a resort within 1 hour of Stanford, California. The resort featured conference rooms for didactic sessions, indoor and outdoor lounge space, a swimming pool, fitness center and spa, and hiking trails. Following the 2-day workshop, a series of 3 optional evening follow-up sessions were held in a hotel conference room near Stanford, with dinner provided.

### Study Population

Stanford Medicine faculty physicians invited to participate in the immediate intervention group, henceforth referred to as the *intervention group*, and those invited to participate in the delayed intervention control group, henceforth referred to as the *control group*, had at least 1 of the following characteristics: (1) worked in a department or division hypothesized to have experienced the greatest COVID-19–related work burden and/or (2) worked in a department or division with unfavorable IWPR scores on an institution-wide survey. The eligible departments or divisions were subsequently grouped by size so that roughly half of the faculty were invited to the intervention group (ie, invited to participate in the fall 2022 retreat) and half invited to the control group (ie, invited to participate in the spring 2023 retreat). Benefits-eligible faculty from the following divisions or departments were invited to participate in the intervention group: emergency medicine, otolaryngology, hospital medicine, medical oncology, medical hematology, pediatric infectious diseases, pediatric hospital medicine, and pediatric critical care medicine. Faculty from the following divisions or departments were recruited for the control group: anesthesiology, blood and marrow transplantation, neurology, nephrology, pediatric immunology, and neonatology. The department of emergency medicine and the divisions of hospital medicine, pediatric infectious diseases, pediatric hospital medicine, and pediatric critical care medicine were selected because of the effect of the COVID-19 pandemic; the other departments and divisions were selected because of unfavorable department or division IWPR scores on the institutional survey.

### Intervention Procedures

**Figure 1.**
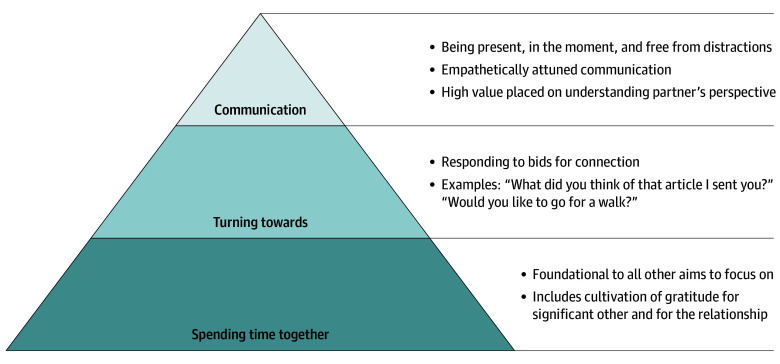
Diagram Illustrating Important Concepts for Strengthening Relationships These themes and concepts provided scaffolding for the didactic content at the workshop weekend and subsequent follow-up sessions.

The first 2-day workshop occurred in October 2022. The purpose of the 2-day workshop itself was to (1) provide physicians and their partners time and space away from the constraints of daily life, to foster connection and relaxation; (2) provide a mix of didactic and reflective education meant to strengthen personal relationships, using positive psychology methods (including discussion prompts that cultivated gratitude, exercises that focused on building on strengths, and tips for collaborative communication)^21,22^; and (3) allow faculty and their partners to meet and connect with other couples with similar experiences, to both validate their own experience and learn from others. As such, the workshop included a mix of “structured” time in didactic or facilitated discussion sessions, “unstructured” free time meant to be spent as a couple (with a suggested gratitude exercise), and loosely styled social activities, such as optional salsa making or guided hiking, as well as discussion prompts left on lunch tables. Interactive didactic sessions included discussion of factors that are cultivated in physician professional culture^23,24^ that may lead to challenges in close personal relationships outside of work.^25^ Strategies to mitigate the potential adverse IWPR of these factors were also discussed.^26,27^ Couples also participated in facilitated breakout sessions that addressed the intricacies of work-life integration and provided a forum to discuss potential work-home conflict and set and commit to goals.^3^ After the workshop weekend, a series of 3 optional evening follow-up sessions were offered to the physician faculty and their partners. These sessions were held over dinner at a local hotel and included a mix of didactic content and reflective discussion. The purpose of these sessions was to reinforce and expand on the content first presented during the weekend retreat (Figure 1). For additional information on workshop and follow-up content, please see eFigures 1 and 2 in Supplement 1.

### Data Collection

Both the intervention group and the control group were assessed at multiple intervals. IWPR was measured with a survey asking 4 questions about how work had affected personal relationships during the past year, and the responses included not at all true, somewhat true, moderately true, very true, and completely true (with scores assigned as 0, 1, 2, 3, and 4, respectively); the full scales and scoring regimens are included in Supplement 1. Each assessment was identical and included the following scales, which have been published previously: adverse IWPR,^14,15^ self-valuation,^28^ and the professional fulfillment index subscales measuring work exhaustion, interpersonal disengagement, and overall burnout^29^ (eFigure 3 in Supplement 1).

**Figure 2.**
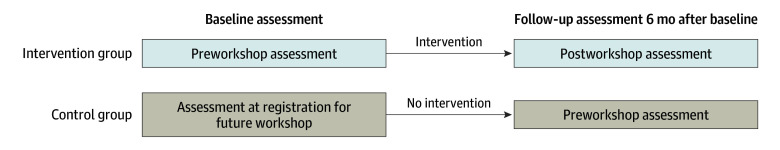
Timing of Assessments for Intervention and Control Groups

The intervention group completed an assessment immediately before the intervention in October 2022 and 6 months after the intervention (Figure 2) in April 2023. Faculty participants who completed the postworkshop assessment received a $50 gift card to a local restaurant. The control group completed their baseline assessment in November 2022 at the time of initial registration (ie, at a similar time point as the preworkshop assessment for the intervention group) and 6 months later in early May 2023 (ie, shortly before they participated in the workshop and at a similar time point as the 6-month postintervention assessment for the intervention group).

### Statistical Analysis

The data were analyzed from June 14, 2024, to October 1, 2024. Participant scores from IWPR, burnout, and self-valuation were normalized on a scale from 0 to 10 to facilitate comparative interpretation; for IWPR and burnout, lower scores are favorable; for self-valuation, a higher score is favorable. Baseline well-being survey measures were compared between the intervention and control groups using a 2-sample *t* test on all available data. To compare baseline and 6-month responses for both the intervention and control groups, only individuals from each group who completed surveys at both time points were included in the paired analysis. Baseline and 6-month well-being survey measures were summarized using descriptive statistics for both cohorts. A paired *t* test was used to examine the difference in well-being measures before and after the test. The Cohen *d* within-group effect sizes were reported.

Linear mixed-effects regression, using all available data, with an interaction term between time point and group was used to examine the association between changes in well-being measures and whether the responders were part of the intervention or control cohort. All data points were included for this intention-to-treat analysis, regardless of whether they could be paired across pretest and posttest surveys. The Glass *d* effect size was reported, which is the mean difference between groups divided by the baseline SD.^30^ The mean difference between groups was determined by the group-by-time interaction effect from the mixed-effects model analysis. To determine whether attendance at 1 or more optional evening follow-up sessions was associated with changes in well-being, an additional analysis was carried out by adding the number of follow-up sessions attended to the mixed-effects model as a covariate. Statistical significance was set at 2-tailed *P* < .05. All analyses were performed in R software, version 4.3.2 (R Project for Statistical Computing).

## Results

A total of 60 faculty members from the intervention group departments or divisions registered for the couples’ workshop with their partners; 47 completed the baseline assessment immediately before the workshop, and ultimately 44 couples participated in the workshop weekend. Of the 47 faculty members who completed the baseline assessment, 22 (46.8%) were women. Thirteen faculty members were in the department of emergency medicine, 11 in the department of medicine, 1 in the department of neurology, and 22 in the department of pediatrics. Sixteen couples who initially registered did not attend for a variety of reasons, including lack of childcare and illness, including 3 couples who completed the baseline assessment and canceled at the last minute (ie, between taking the assessment and the start of the workshop). Paired baseline and 6-month assessment data were available for 38 (80.9%) of the 47 faculty members who completed the baseline assessment. A total of 69 faculty members from the control group departments or divisions registered to participate, of whom 40 (57.9%) were women. Thirty-six faculty members were from the department of anesthesiology, 5 from the department of medicine, 21 from the department of neurology, and 7 from the department of pediatrics. Paired baseline and 6-month baseline and 6-month assessment data were available for 53 of 69 (76.8%) of these faculty members (eFigure 4 in Supplement 1). Between the baseline and 6-month assessments, 16 couples in the control group canceled for reasons similar to those reported for the intervention group. There were no statistically significant differences in the sex composition of the intervention and control groups (46.8% vs 57.9% women; *P* = .32).

Baseline assessment scores for each group are presented in Table 1; there was no significant difference in baseline IWPR, self-valuation, or burnout scores between the 2 groups. Baseline and 6-month assessment scores for the intervention group and control group are presented in Table 2. Six months after the intervention, faculty participants in the intervention group had a mean (SD) adverse IWPR score that was 1.59 (2.66) points (Cohen *d* = 0.54 [95% CI, 0.23-0.85]) lower (3.34 vs 4.93; *P* < .001 [lower score favorable]), burnout scores that were 1.22 (1.47) points (Cohen *d* = 0.68 [95% CI, 0.39-0.98]) lower (2.55 vs 3.78; *P* < .001 [lower score favorable]), and self-valuation scores that were 1.25 points (2.09) (Cohen *d* = 0.68 [95% CI, –0.97 to –0.25]) higher (5.95 vs 4.70; *P* < .001 [higher score favorable]). No significant differences in IWPR, burnout, or self-valuation scores were detected between the baseline and 6-month assessment in the control group.

**Table 1.** Baseline Outcome Measures

Outcome measure	Mean (SD) score^a^		*P* value
	Intervention group (n = 47)	Control group (n = 69)	
Adverse impact of work on personal relationships	5.01 (3.10)	5.22 (2.37)	.69
Self-valuation	4.53 (2.05)	4.12 (1.91)	.28
Overall burnout	3.83 (1.84)	3.46 (1.75)	.28
Interpersonal disengagement	3.26 (2.15)	2.88 (1.82)	.31
Work exhaustion	4.69 (1.85)	4.34 (2.07)	.35

^a^ Scale from 0 to 10 (for impact of work on personal relationships and burnout, lower scores are favorable; for self-valuation, a higher score is favorable), with at least 75% response rate.

**Table 2.** Baseline and 6-Month Assessment for Intervention and Control Groups

Outcome measure^a^	Intervention group					Control group				
	Mean (SD) score		Mean difference (SD)^b^	*P* value	Cohen *d* effect size (95% CI)	Mean (SD) score		Mean difference (SD)^b^	*P* value	Cohen *d* effect size (95% CI)
	Baseline	6-mo Follow-up				Baseline	6-mo Follow-up			
Adverse impact of work on personal relationships	4.93 (3.24)	3.34 (2.56)	1.59 (2.66)	<.001	0.54 (0.23 to 0.85)	5.42 (2.30)	4.96 (2.80)	0.46 (1.84)	.07	0.17 (−0.02 to 0.37)
Self-valuation	4.70 (2.12)	5.95 (1.95)	−1.25 (2.09)	<.001	−0.61 (−0.97 to −0.25)	3.97 (1.75)	4.34 (1.92)	−0.37 (1.76)	.14	−0.2 (−0.46 to 0.06)
Overall burnout	3.78 (1.96)	2.55 (1.35)	1.22 (1.47)	<.001	0.68 (0.39 to 0.98)	3.59 (1.69)	3.57 (1.81)	0.02 (1.35)	.90	0.01 (−0.20 to 0.22)
Interpersonal disengagement	3.15 (2.26)	2.06 (1.49)	1.09 (1.77)	<.001	0.53 (0.23 to 0.83)	2.94 (1.82)	3.04 (1.80)	−0.10 (1.43)	.60	−0.06 (−0.27 to 0.16)
Work exhaustion	4.72 (1.98)	3.29 (1.64)	1.43 (1.71)	<.001	0.78 (0.43 to 1.12)	4.56 (1.96)	4.35 (2.09)	0.21 (1.61)	.34	0.1 (−0.11 to 0.32)

^a^ Scale from 0 to 10 (for impact of work on personal relationships and burnout, lower scores are favorable; for self-valuation, a higher score is favorable), with at least 75% response rate (37 participants for impact of work on personal relationships and 38 participants for all other measures for the intervention group; 53 participants for all measures for the delayed intervention control group).

^b^ Paired *t* test.

The mixed-effects model comparing the intervention group with the control group is presented in Table 3. Being in the intervention group was associated with an improvement in IWPR (Glass *d* = −0.45; *P* = .01) and self-valuation (Glass *d* = 0.47; *P* = .02) at the 6-month time point relative to the delayed intervention control group. Being in the intervention group was also associated with improvement in overall burnout (Glass *d* = −0.70; *P* < .001) and burnout subscale scores for interpersonal disengagement (Glass *d* = −0.65; *P* < .001) and work exhaustion (Glass *d* = −0.64; *P* < .001) at the 6-month time point. Additional analysis of the 18 faculty members who participated in 1 or more of the 3 optional evening follow-up sessions did not show a statistically significant difference in any domain relative to intervention participants who did not attend any follow-up sessions.

**Table 3.** Intervention vs No Intervention (N = 116)

Measure	Controlled group difference^a^	*P* value for significance	Baseline SD	Glass *d* effect size (95% CI)^b^
Adverse impact of work on personal relationships	−1.25	.01	2.75	−0.45 (−0.12 to −0.79)
Self-valuation	0.97	.02	2.05	0.47 (0.09 to 0.86)
Overall burnout	−1.24	<.001	1.76	−0.70 (−0.37 to −1.03)
Interpersonal disengagement	−1.21	<.001	1.87	−0.65 (−0.30 to −1.00)
Work exhaustion	−1.28	<.001	1.99	−0.64 (−0.30 to −0.99)

^a^ Based on linear mixed-effects regression model with an interaction between time point and cohort.

^b^ Mean difference between groups divided by the baseline SD.

## Discussion

We report here the associations of an organization-based pilot program for physicians and their partners with the adverse impact of work on physicians’ personal relationships. Across all domains measured (ie, IWPR, burnout, and self-valuation), the intervention group had a meaningful effect size and statistically significant improvements at the 6 month time point relative to the control group. The effect size changes in these dimensions ranged from 0.4 to 0.7, suggesting small to moderate intervention effects.

The workshop content was designed specifically for physicians and focused on personal traits and behaviors cultivated by the professional culture, as well as strategies to foster work-life integration. Based on published literature, these issues likely contribute to the adverse IWPR.^2,25,31^ We hypothesized that bringing an awareness to these traits and behaviors, providing structure and space for reflection both alone and with their partner, and providing the opportunity to evaluate and set goals with respect to work-life integration would help mitigate the adverse IWPR. Although the educational environment and content delivered through the workshop weekend and follow-up sessions were specifically designed to improve the outcome dimensions that improved in the paired assessment (ie, IWPR, burnout, and self-valuation), more study is needed to determine the effect of each component of the intervention and how they may be enhanced.

These data provide evidence that this type of organizationally sponsored couples workshop may be an effective tool at helping to mitigate the adverse impact of work on physicians’ personal relationships. However, work at the individual level alone is likely insufficient to mitigate the greater adverse IWPR among physicians relative to the population.^14^ Simultaneous changes to the professional culture of medicine and the practice structure that contributes to the adverse IWPR and burnout are essential.^24^ Specifically, character traits and practices cultivated by our professional culture, such as perfectionism, deferred gratification, preoccupation with problem solving, and deferral of self-care, can all lead to challenges in our personal relationships, either directly or indirectly.^25,31,32^ Efforts to bring awareness to and shift these cultural norms may be helpful.^23^ For example, institutional or national efforts to emphasize our shared humanity, interventions to improve self-valuation (eg, professional coaching), and promoting a workplace culture that is not only permissive but also encouraging of self-care activities and boundary setting can all help promote personal relationships.^1,17,18,23,24^ Workplace systems that may mitigate the IWPR include realistic and sustainable workloads, ample parental and family leave policies with opportunities for ramp-up on return to work, flexible work arrangements (including remote and hybrid work), scheduling of meetings and other activities during business hours, predictability of scheduling, adequate cross-coverage and team-based care, and protection of vacation time.^33,34,35^ These workplace structures and boundaries can promote relationship health by allowing physicians to be more physically and mentally present in their relationships and to potentially carry less emotional and physical fatigue from work. Evidence indicates that attention to these dimensions of workplace structure is essential for health care organizations to provide a high quality of care and an optimal patient experience.^15,36^

### Limitations

These findings should be considered in the context of several limitations. First, although the characteristics assessed at baseline between groups appeared balanced, the quasi-experimental design may not have resulted in equivalent groups in unmeasured confounding variables, including differences in departmental culture and work structure of the departments invited. However, there were no significant baseline group differences in outcome variables. Second, this was a pilot intervention at a single academic medical center with a relatively small sample size. Although our results demonstrated improvements 6 months after the intervention, it is unclear how long the observed benefits persist and what type of ongoing support may be necessary to preserve the benefits observed beyond 6 months. In addition, although we used standardized measures of well-being to assess long-term outcomes, we are unable to evaluate the contribution of the individual didactic and reflection elements that made up the workshops, to determine the contribution of each component to the improvements observed. Although there was clearly a financial expense associated with this organizationally sponsored workshop, the meaningful effect size improvement in occupational well-being outcome measures shown to be associated with the quality of care and patient experience suggests that it may be a high-value investment.^15,36^ In addition, the moderate effect size improvement in physician burnout at 6 months may result in cost savings to the organization that far outstrip the cost of the workshop itself, the cost of which equated to less than 1% of the annual compensation for the average physician at our center.^37,38,39,40^ Furthermore, because a limited number of faculty participate in the retreats, the overall cost to an organization is far less than 1% of total physician compensation. This intervention focused on 1 specific type of relationship: the physician’s relationship with their spouse or partner. Future interventions should seek to help physicians focus on and strengthen other important relationships in their lives (eg, friendships, relationships with children, and other family members [parents and siblings]).

## Conclusions

In this cohort study of an organizationally sponsored intervention consisting of a couples’ workshop designed to mitigate the adverse IWPR for physicians, participation was associated with statistically significant and meaningful effect size improvements in IWPR, burnout, and self-valuation. These data provide evidence that this approach may be an effective strategy to reduce the adverse IWPR, mitigate burnout, and improve physician well-being. Future studies are needed to replicate these findings, enhance impact, and determine how to sustain the impact of such interventions.

## Notes

Supplement 1. **eFigure 1.** Weekend Agenda and Session Description **eFigure 2.** Weekend Content Highlights **eFigure 3.** Survey Measures and Scoring **eFigure 4.** Flow Diagram of Eligible and Enrolled Faculty

Supplement 2.
Data Sharing Statement
