# Supplementary material for: Implementation of an Organization-Based Couples Health Promotion Program to Improve Physician Well-Being
Source: JAMA Netw Open. 2025 Apr 4;8(4):e253218. doi: 10.1001/jamanetworkopen.2025.3218 (PMC11971666; doi:10.1001/jamanetworkopen.2025.3218)
Supplement: Supplement 1. — eFigure 1. Weekend Agenda and Session Description eFigure 2. Weekend Content Highlights eFigure 3. Survey Measures and Scoring eFigure 4. Flow Diagram of Eligible and Enrolled Faculty [file jamanetwopen-e253218-s001.pdf]

Supplemental Online Content

Gold JM, Shanafelt TD, Wang H, et al. Implementation of an organization-based couples health promotion program to improve physician well-being. *JAMA Netw Open*. 2025;8(4):e253218. doi:10.1001/jamanetworkopen.2025.3218

eFigure 1. Weekend Agenda and Session Description
eFigure 2. Weekend Content Highlights
eFigure 3. Survey Measures and Scoring
eFigure 4. Flow Diagram of Eligible and Enrolled Faculty

This supplemental material has been provided by the authors to give readers additional information about their work.

© 2025 Gold JM et al. *JAMA Network Open*.

eFigure 1: Weekend Agenda and Session Description

Time	Activity	Participants	Location	Themes
Saturday				
8:30-9:15 AM	Breakfast	All	Sunset Restaurant and Patio	
9:15-9:30 AM	Check-in with Staff	All	Seascape Ballroom	
9:30-11:00 AM	Opening Session	All	Seascape Ballroom	Re-establishing the importance of committing time and energy to our personal relationships; gaining a better understanding of ourselves and each other
11:00 AM-12:30 PM	Independent Couple Time: Fostering Reconnection	Couples	Suggested on-property activities	Theme: Gratitude cultivation
12:30-1:30 PM	Lunch with Table Discussion	All	Sunset Restaurant and Patio	
1:40-2:40 PM	Workshops: Work-Life Integration	All	Seascape Ballroom & Breakout Rooms	Reflecting on the nuts and bolts of daily life at home and at work; re-negotiating division of labor
3:00-6:00 PM	Independent Couple Time: Connection/Relaxation	Couples/Small Groups	Suggested on- and off-property activities	
6:00-8:00 PM	Dinner	All	Sunset Restaurant and Patio	
8:00-10:00 PM	Time Together at Firepits with S'mores	All (optional)	Sunset Patio	
Sunday				
8:30-9:30 AM	Breakfast and Date Planning Fair	All	Seascape Ballroom	Planning for time together

© 2025 Gold JM et al. *JAMA Network Open*.

9:30-11:00 AM	Reflection and Goal Setting: Prioritizing Time Together	All	Seascape Ballroom	Strengthening the way we prioritize time together
11:00 AM	Retreat Concludes and Hotel Check-Out			

© 2025 Gold JM et al. *JAMA Network Open*.

eFigure 2: Weekend Content Highlights

Guiding Design Principles

Based in positive psychology
Couples need a mix of structured didactic/facilitated discussion time, as well as unstructured time for connection with each other, as well as social connection
Learning from others with a shared experience may be both validating and inspiring
Having time and space away from the stressors of daily life can help facilitate productive discussion about building the "elements of daily life."

Stanford MEDICINE | WellMD WellPhD

Content Highlights: Four Physician Strengths and Associated Growth Opportunities

- Deferred Gratification
- Ability to Find and Solve Problems
- Constant Drive to Do, Serve, and Accomplish More
- Exacting Standards for Oneself

© 2025 Gold JM et al. *JAMA Network Open*.

Content Highlights: Work-Life Integration Workshop

- Dual purpose
  - Provide structure for couples to reflect on and examine individual and shared work-life integration goals
  - Promote discussion among couples in a similar "phase of life."

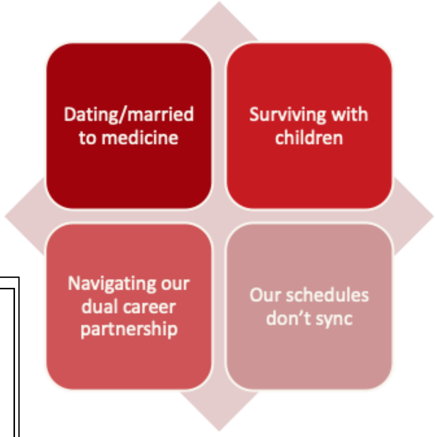

Couples chose one of four workshop groupings described in the boxes to the right

Stanford MEDICINE | WellMD WellPhD

© 2025 Gold JM et al. *JAMA Network Open*.

eFigure 3: Survey Measures and Scoring

Burnout (emotional exhaustion and interpersonal disengagement)[1]

To what degree have you experienced the following?

**During the past two weeks, I have felt…**	**Not At All**	**Very Little**	**Moderately**	**A Lot**	**Extremely**
Scoring	*0*	*1*	*2*	*3*	*4*
**A sense of dread when I think about work I have to do**	[ ]	[ ]	[ ]	[ ]	[ ]
**Physically exhausted at work**	[ ]	[ ]	[ ]	[ ]	[ ]
**Lacking in enthusiasm at work**	[ ]	[ ]	[ ]	[ ]	[ ]
**Emotionally exhausted at work**	[ ]	[ ]	[ ]	[ ]	[ ]

**During the past two weeks, my job has contributed to me feeling…**	**Not At All**	**Very Little**	**Moderately**	**A Lot**	**Extremely**
Scoring	*0*	*1*	*2*	*3*	*4*
Less empathetic with my patients	[ ]	[ ]	[ ]	[ ]	[ ]
Less empathetic with my colleagues	[ ]	[ ]	[ ]	[ ]	[ ]
Less sensitive to others' feelings/emotions	[ ]	[ ]	[ ]	[ ]	[ ]
Less interested in talking with my patients	[ ]	[ ]	[ ]	[ ]	[ ]
Less connected with my patients	[ ]	[ ]	[ ]	[ ]	[ ]
Less connected with my colleagues	[ ]	[ ]	[ ]	[ ]	[ ]

Self-Valuation[2]

How often have you experienced the following?

**During the past two weeks…**	**Never**	**Rarely**	**Sometimes**	**Often**	**Always**
Scoring	*4*	*3*	*2*	*1*	*0*
**a. When I made a mistake, I felt more self-condemnation than self-encouragement to learn from the experience**	[ ]	[ ]	[ ]	[ ]	[ ]
**b. I was less compassionate with myself than I was with others**	[ ]	[ ]	[ ]	[ ]	[ ]
**c. I put off taking care of my own health due to time pressure**	[ ]	[ ]	[ ]	[ ]	[ ]
**d. Taking care of my needs seemed incompatible with taking care of my patients' needs**	[ ]	[ ]	[ ]	[ ]	[ ]

Negative Impact of Work on Relationships[3]

How has your job affected your personal relationships during the past year?

**In the past year, my job has…**	**Not At All True**	**Somewhat True**	**Moderately True**	**Very True**	**Completely True**

[1] Trockel M, Bohman B, Lesure E, et al. A Brief Instrument to Assess Both Burnout and Professional Fulfillment in Physicians: Reliability and Validity, Including Correlation with Self-Reported Medical Errors, in a Sample of Resident and Practicing Physicians. Acad Psychiatry J Am Assoc Dir Psychiatr Resid Train Assoc Acad Psychiatry. 2018;42(1):11-24. doi:10.1007/s40596-017-0849-3

[2] Trockel MT, Hamidi MS, Menon NK, et al. Self-valuation: Attending to the Most Important Instrument in the Practice of Medicine. Mayo Clin Proc. 2019;94(10):2022-2031. doi:10.1016/j.mayocp.2019.04.040

[3] Trockel J, Bohman B, Wang H, Cooper W, Welle D, Shanafelt TD. Assessment of the Relationship Between an Adverse Impact of Work on Physicians' Personal Relationships and Unsolicited Patient Complaints. Mayo Clin Proc. 2022;97(9):1680-1691. doi:10.1016/j.mayocp.2022.03.005

© 2025 Gold JM et al. *JAMA Network Open*.

Scoring	*0*	*1*	*2*	*3*	*4*
**a. Made it harder for me to nurture <u>existing</u> personal relationships**	[ ]	[ ]	[ ]	[ ]	[ ]
**b. Made it harder for me develop <u>new</u> meaningful personal relationships**	[ ]	[ ]	[ ]	[ ]	[ ]
**c. Contributed to conflict in my personal relationship(s)**	[ ]	[ ]	[ ]	[ ]	[ ]
**d. Contributed to me feeling more isolated or detached from the people who are most important to me**	[ ]	[ ]	[ ]	[ ]	[ ]

© 2025 Gold JM et al. *JAMA Network Open*.

eFigure 4: Flow diagram of eligible and enrolled faculty

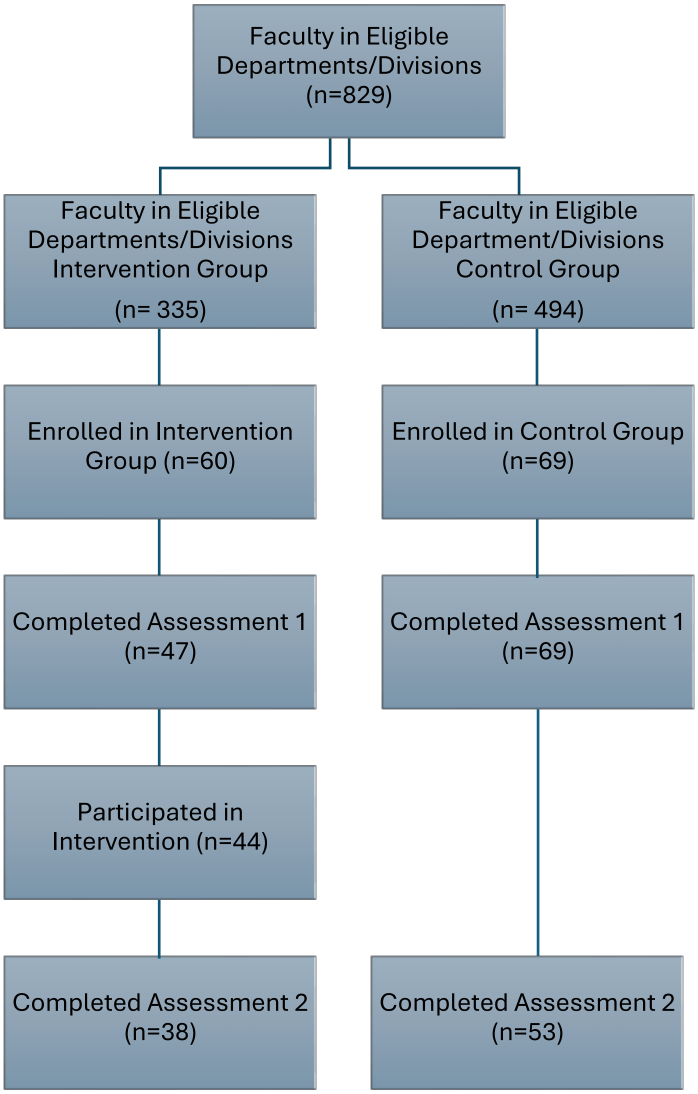

© 2025 Gold JM et al. *JAMA Network Open*.
