# Supplementary material for: Implementation of an Organization-Based Couples Health Promotion Program to Improve Physician Well-Being
Source: JAMA Netw Open. 2025 Apr 4;8(4):e253218. doi: 10.1001/jamanetworkopen.2025.3218 (PMC11971666; doi:10.1001/jamanetworkopen.2025.3218)
Supplement: Supplement 2. — Data Sharing Statement [file jamanetwopen-e253218-s002.pdf]

Data Sharing Statement

Gold. Implementation of an Organization-Based Couples Health Promotion Program to Improve Physician Well-Being. *JAMA Netw Open*. Published April 04, 2025. doi:10.1001/jamanetworkopen.2025.3218

Data
Data available: No
